# The cerebrospinal fluid immunoglobulin transcriptome and proteome in neuromyelitis optica reveals central nervous system-specific B cell populations

**DOI:** 10.1186/s12974-015-0240-9

**Published:** 2015-01-28

**Authors:** Markus C Kowarik, Monika Dzieciatkowska, Scott Wemlinger, Alanna M Ritchie, Bernhard Hemmer, Gregory P Owens, Jeffrey L Bennett

**Affiliations:** Department of Neurology, 12700 E. 19th Ave, Box B-182, Aurora, CO 80045 USA; Department of Ophthalmology, Neuroscience Program, Denver, CO USA; Department of Biochemistry, University of Colorado Denver, Denver, CO USA; Department of Neurology, TU-München, Klinikum Rechts der Isar, Munich, Germany; Munich Cluster for Systems Neurology (SyNergy), Munich, Germany

**Keywords:** Neuromyelitis optica, Aquaporin-4, Immunoglobulin G, Proteome, Transcriptome, Autoantibodies

## Abstract

**Background:**

Neuromyelitis optica (NMO) is a severe demyelinating disorder of the central nervous system (CNS) associated with the presence of an autoimmune antibody response (AQP4-IgG) against the water channel aquaporin-4 (AQP4). It remains unclear whether pathologic AQP4-IgG in the CNS is produced entirely by peripheral plasma cells or is generated in part by infiltrating B cells. To determine the overlap of AQP4-IgG idiotypes between the CNS and periphery, we compared the immunoglobulin G (IgG) transcriptome of cerebrospinal fluid (CSF) plasmablasts with the CSF and serum IgG proteomes in 7 AQP4-seropositive NMO patients following exacerbation.

**Methods:**

CSF variable region Ig heavy- (VH) and light-chain (VL) transcriptome libraries were generated for each patient from CSF plasmablasts by single cell sorting, reverse transcriptase polymerase chain reaction (RT-PCR), and DNA sequencing. Recombinant antibodies were generated from clonally expanded, paired VH and VL sequences and tested for AQP4-reactivity by cell-binding assay. CSF and serum IgG fractions were searched for sequences that matched their respective CSF IgG transcriptome. Matching peptides within the same patient’s CSF and serum IgG proteomes were also identified.

**Results:**

In each NMO patient, we recovered CSF IgG VH and VL sequences that matched germline-mutated IgG protein sequences from the patient’s CSF and serum IgG proteomes. Although a modest variation was observed between patients, the overlap between the transcriptome and proteome sequences was found primarily, but not exclusively, within the CSF. More than 50% of the CSF IgG transcriptome sequences were exclusively found in the CSF IgG proteome, whereas 28% were found in both the CSF and blood IgG proteome, and 18% were found exclusively in the blood proteome. A comparable distribution was noted when only AQP4-specific IgG clones were considered. Similarly, on average, only 50% of the CSF IgG proteome matched corresponding peptide sequences in the serum.

**Conclusions:**

During NMO exacerbations, a substantial fraction of the intrathecal Ig proteome is generated by an intrathecal B cell population composed of both novel and peripherally-derived clones. Intrathecal CSF B cell clones may contribute to NMO disease exacerbation and lesion formation and may be an important target for preventative therapies.

## Background

Neuromyelitis optica (NMO) is a severe central nervous system (CNS) autoimmune disorder with predilection for the optic nerves and spinal cord [1]. Specific to the disease is an autoimmune population of B cells that produces autoantibodies (AQP4-IgG) against the water channel aquaporin-4 (AQP4) [2-5]. Although controversial, serum and cerebrospinal fluid (CSF) titers of AQP4-IgG have been shown to correlate loosely with clinical disease activity and disability in affected individuals. Serum AQP4-IgG titers are increased at the nadir of exacerbations, and higher serum and CSF AQP4-IgG titers are observed in patients with severe lesions [6,7]. Conversely, clinical improvement has been correlated with a decrease in CSF AQP4-IgG titers [8].

The relationship between serum and CSF titers of AQP4-IgG and the presence of intrathecal AQP4-IgG production, however, remains unclear. Several studies have suggested that AQP4 autoantibodies are passively transferred from serum to CSF [6,8-10]. In contrast, other studies have documented intrathecal synthesis of AQP4-IgG [3,8,11]. Klawiter and colleagues [12] also reported three cases in which AQP4-IgG was restricted to the CSF, but this study remains controversial due to methodological issues. Using standard clinical laboratory methods, CSF oligoclonal IgG bands have been infrequently observed in NMO patients; and if present, the level of intrathecal IgG synthesis was low, transient, and restricted to acute relapses [9,11,13].

The identification of clonally expanded CSF plasmablasts expressing AQP4-IgG in an NMO patient [3] suggests that intrathecal B cell populations may contribute to CSF AQP4-IgG and disease pathogenesis. To study the origin of CSF AQP4-IgG, we compared the immunoglobulin G (IgG) transcriptome of CSF plasma cells with the IgG CSF and serum proteomes in seven AQP4-seropositive NMO patients. The overlapping sequences indicate that intrathecal antibody production contributes measurably to the generation of CSF AQP4-IgG.

## Methods

### Patients

AQP4-seropositive NMO or NMO spectrum disease (NMOSD) patients experiencing an acute exacerbation were recruited at the University of Colorado (ON10-03; ON09-03; ON10-01; ON07-05; ON08-08; ON11-04) and the Technische Universität München (TUM09-527). CSF analysis was performed within 84 days of symptom onset (median: 28 days; range 1 to 84 days). Diagnoses were made according to published criteria [14,15]. Patient CSF was obtained as a part of their standard clinical evaluation; informed consent was obtained prior to study participation. The ethics committee of the University of Colorado Denver and Technische Universität München approved the scientific use of CSF and serum samples. Biologic samples were processed and stored according to consensus guidelines [16]. As prior work has reported decreased serum AQP4-IgG titers following treatment with rituximab and methylprednisolone [7], CSF was collected prior to or 4 weeks following steroid administration (ON07-5; ON08-8). Patient TUM09-527 was treated with rituximab; all other patients were untreated at the time of exacerbation. Table 1 shows the CSF parameters and the calculated AQP4 antibody index (AQP4-AI). AQP4-AI was determined as described previously [17]. No patient received intravenous immunoglobulin (IVIG) treatment, had a chronic infection, or was noted to have a monoclonal gammopathy by serum electrophoresis or proteomic analysis.

**Table 1:** **Results of routine cerebrospinal fluid (CSF) examination**

	**Normal range**	**ON10-03**	**ON09-03**	**ON10-01**	**ON07-05**	**ON08-08**	**ON11-04**	**TUM09-527**
**Cell count (cells/μl)**	*0 to5*	4	9^a^	16^a^	19^a^	5	2	15^a^
**Albumin serum**	*3,500 to5,200*	4,140	4,330	4,300	4,030	4,100	3,530	3,900
**(mg/dl)**								
**Albumin CSF**	*0 to 35*	32	23	20	38^a^	24	8	38^a^
**(mg/dl)**								
**Albumin Quotient**	*0.0 to 9.0*	7.72	5.31	4.65	9.43^a^	5.85	2.27	9.74^a^
*(x10* ^*3*^ *)*								
**IgG serum**	*768 to 1,632*	462	2,570^a^	634	1,460	2,060^a^	1,210	965
**(mg/dl)**								
**IgG CSF**	*0.0 to 6.0*	4.1	6.3^a^	1.7	6.8^a^	4.9	1.4	4.7
**(mg/dl)**								
**IgG Quotient**	*0.0 to 7.8*	8.87^a^	2.45	2.68	4.65	2.38	1.16	4.87
*(x10* ^*3*^ *)*								
**IgG Intrathecal Fraction (%)**	*NA*	34	0	0	0	0	0	0
**IgG Index**	*0.28 to 0.66*	1.15^a^	0.46	0.58	0.49	0.41	0.51	0.51
**OCBs**	*-*	+	-	-	-	-	-	-
**AQP4 Index**	*(<1.5)*	1.57^a^	0.56	1.45	2.0^a^	0	0	0.86
**(AQP4-AI)**								

^a^Indicates abnormal values; NA, not available.

CSF erythrocytes averaged 2.3 cells/μl (range 0 to 11 cells/μl).

### Single cell analysis of B cells, antibody production and AQP4 reactivity

CSF IgG variable region heavy- (VH) and light-chain (VL) sequences were recovered from CD19-CD138+ plasmablasts by single cell fluorescent-activated cell sorting (FACS), reverse transcriptase PCR (RT-PCR), and DNA sequencing as described previously [18]. Recombinant antibodies were produced in HEK293 cells (Invitrogen, Carlsbad, CA, USA; R620-07) as described previously [3] and reactivity was tested via a quantitative cell-binding immunofluorescence assay using a U87MG permanent cell line expressing M23-AQP4 [19].

### Mass spectrometry of CSF and serum IgG

CSF and serum IgG (0.5 to 2.0 ml) was applied to protein A sepharose columns (GE Healthcare, Pittsburgh, PA, USA) and purified according to the manufacturer’s instructions. Protein concentrations were determined by bicinchoninic acid (BCA) Protein-Assay (Pierce, Thermo Fisher, Waltham, MA, USA), and the purity analyzed by sodium dodecyl sulfate polyacrylamide electrophoresis. Excised heavy- and light-chain gel pieces were destained in ammonium bicarbonate/50% acetonitrile (ACN) and dehydrated in 100% acetonitrile. Disulfide bonds were reduced by dithiothreitol, and cysteine residues were alkylated with iodoacetamide. Heavy-chain proteins were digested with trypsin, light chains with trypsin and *S. aureus* V8 protease (Glu-C). Following digestion, the tryptic mixtures were extracted in 1% formic acid/50% acetonitrile). Samples were analyzed on a linear trap quadropole (LTQ) Orbitrap Velos mass spectrometer (Thermo Fisher Scientific, Waltham, MA, USA) coupled to an Eksigent nanoLC-2D system (Framingham, MA, USA) through a nanoelectrospray LC-MS interface using a 90-minute gradient from 6 to 40% ACN. Peptide fragmentation was performed in a higher energy collisional dissociation cell with normalized collision energy of 40%, and tandem mass spectra were acquired in the Orbitrap mass analyzer. Data acquisition was performed using Xcalibur software (version 2.0.6; Waltham, MA, USA).

### Database searching, protein identification

Tandem mass (MS/MS) spectra were converted into mgf files using an in-house script. Mascot (version 2.2; Matrix Science Inc., London, UK) was used to perform database searches against the Swiss-Prot database and a database containing translated CSF B cell transcriptome repertoires. Peptide tolerance was set at ± 15 ppm with an MS/MS tolerance of ± 0.1 Da from spectra. Full trypsin specificity was required and one missed cleavage was allowed; carbamidomethylation on cysteine was defined as a fixed modification; methionine oxidation, N-terminal pyroglutamic acid formation and N-terminal (protein) acetylation were defined as variable modifications in the database search. Scaffold (version4, Portland, OR, USA) was used to validate MS/MS based peptide and protein identifications. Peptide identifications were accepted at a > 95.0% probability, protein identifications at a > 99.0% probability.

### Alignment of IgG transcriptome and proteome sequences

**Figure 1:**
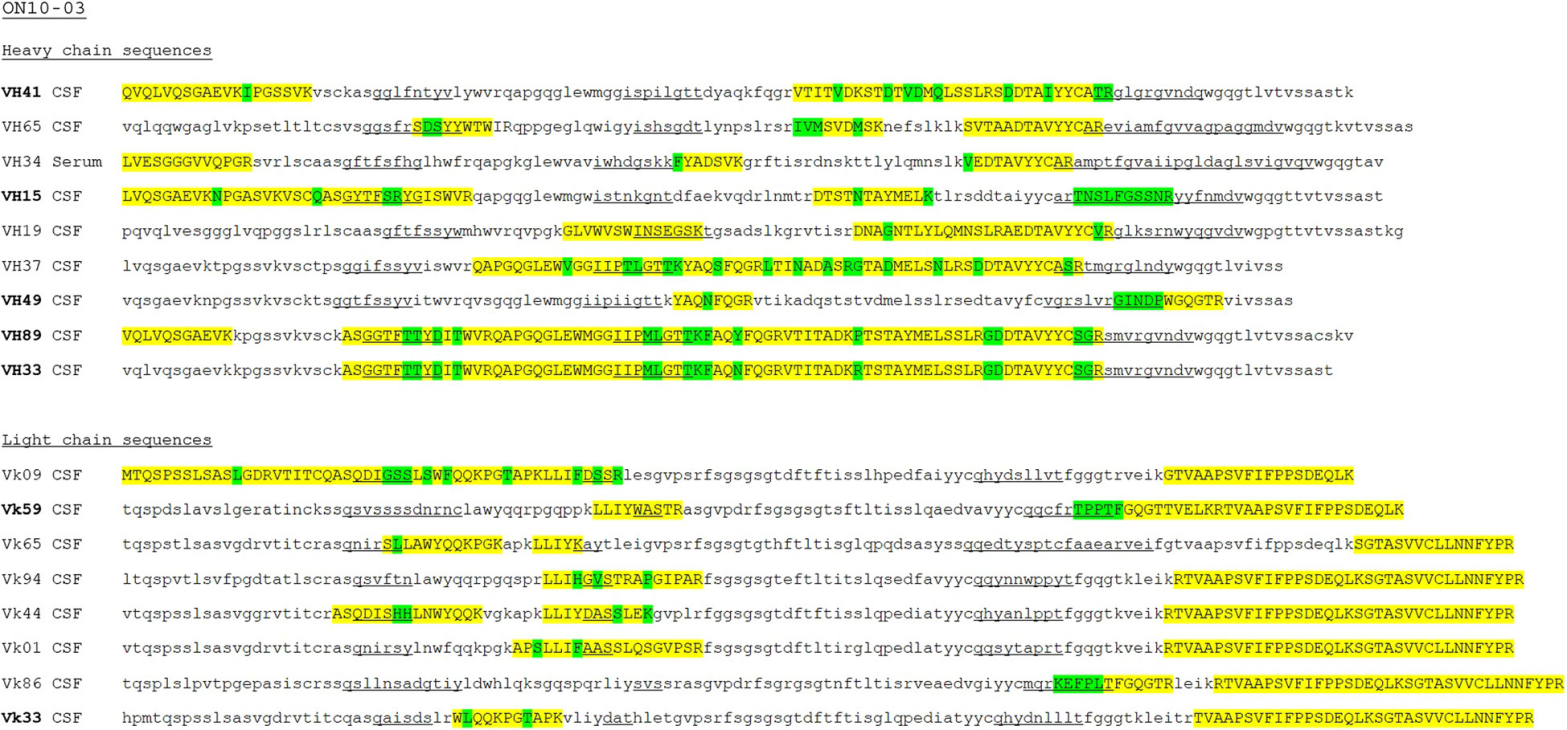
**Alignment of recovered peptides (marked in yellow) to the cerebrospinal fluid (CSF) transcriptome sequences.** Green highlighted letters show mutations from the germline sequence. Aquaporin-4 (AQP4)-specific sequences are marked in bold (for example, **VH33**), CDR1, CDR2 and CDR3 parts are underlined. On average, greater than 80% of the translated CSF transcriptome sequences were identified by 2 or more Ig peptide sequences.

To search for an overlap between IgG transcriptome sequences and IgG proteome peptides, recovered peptides were aligned to the same patient’s transcriptome sequences using Scaffold software (Portland, OR, USA). Peptides were considered unique when they were identical to the somatically mutated cDNA sequence of the VH or VL sequence (Figure 1). To determine the extent of overlap between CSF and blood Ig sequences, peptides were aligned to the human protein database (Swiss-Prot), and matching IgG tested for mutations to the germline using Vbase2 (http://www.vbase2.org/).

## Results

### Analysis of IgG transcriptomes and proteomes

We generated subject-specific variable region VH and VL repertoires from seven NMO and NMOSD patients and in conjunction assessed IgG VH and VL peptide libraries from the patients’ CSF and serum. Using the CSF VH and VL transcriptome repertoires as databases, we examined the serum and CSF IgG proteomes for matching unique sequences defined as peptides perfectly matching somatically-mutated CSF transcriptome sequences (Figure 1). No unique serum or CSF IgG peptides matched CSF transcripts from another NMO patient. On average, 27% (range 15 to 44%) of the unique heavy- and light-chain CSF transcriptome sequences could be recovered in IgG peptides. A slightly higher percentage of transcripts corresponding to known AQP4-specific antibodies (mean 37%; range 0 to 60%) were recovered in the IgG proteomes (Table 2). The efficiency of matching CSF IgG transcripts with peptides in the CSF or serum proteome did not correlate with evidence of blood-CSF barrier disruption, although the maximal recovery of matching peptides occurred in subject ON10-03 who had an elevated IgG index and oligoclonal banding (Table 1). Intrathecal production of AQP4-IgG (AQP4-AI ≥ 1.5) was observed in patients ON10-03 and ON7-05 (Table 1). Patient ON10-01 had an elevated AQP4-AI at 1.45 but the value did not exceed levels indicative of intrathecal synthesis. While treatment of patient TUM09-527 with rituximab may have influenced peripheral antibody production, a comparable number of serum peptides could still be matched to AQP4-specific and nonspecific CSF Ig transcripts. There was no relationship between the timing of the lumbar puncture and the recovery of serum or CSF IgG peptides, although the study may have been underpowered to detect a difference.

**Table 2:** **Fraction of cerebrospinal (CSF) transcriptome sequences identified by matching IgG peptides in serum and CSF**

	**Total transcriptome sequences recovered**		**Transcriptome sequences with matching IgG peptides in CSF only**		**Transcriptome sequences with matching IgG peptides in CSF and serum**		**Transcriptome sequences with matching IgG peptides in serum only**	
**Subject**	**Total**	**AQP4-Specific**	**Total**	**AQP4-Specific**	**Total**	**AQP4-Specific**	**Total**	**AQP4-Specific**
ON10-03	15/34	6/10	14/15	6/6	1/15	0/6	0/15	0/6
	(44%)	(60%)	(93%)	(100%)	(7%)	(0%)	(0%)	(0%)
ON09-03	26/79	6/14	14/26	5/6	11/26	1/6	1/26	0/6
	(33%)	(43%)	(54%)	(83%)	(42%)	(17%)	(4%)	(0%)
ON10-01	17/106	0/9	9/17	NR	7/17	NR	1/17	NR
	(16%)	(0%)	(53%)		(41%)		(6%)	
ON07-05	24/115	6/17	9/24	2/6	11/24	2/6	4/24	2/6
	(21%)	(35%)	(37%)	(33%)	(46%)	(33%)	(17%)	(33%)
ON08-08	18/49	2/3	10/18	0/3	5/18	1/2	3/18	1/2
	(37%)	(66%)	(55%)	(0%)	(28%)	(50%)	(17%)	(50%)
ON11-04	13/86	2/13	1/13	0/2	3/13	1/2	9/13	1/2
	(15%)	(15%)	(8%)	(0%)	(23%)	(50%)	(69%)	(50%)
TUM09-527	11/49	6/16	8/11	5/6	1/11	0/6	2/11	1/6
	(22%)	(38%)	(73%)	(83%)	(9%)	(0%)	(18%)	(17%)
**Mean**	**27%**	**37%**	**53%**	**50%**	**28%**	**25%**	**18%**	**19%**
*95% CI*	*16 to37%*	*15 to- 58%*	*28 to 78%*	*3 to 97%*	*13 to 33%*	*1 to 49%*	*0 to 63%*	*0 to 40%*

*Abbreviations:* *AQP4* aquaporin-4, *CSF* cerebrospinal fluid, *NR* none recovered, *95% CI* 95% confidence interval.

### The overlap of IgG transcriptomes and proteomes is primarily in the CSF

IgG peptides that uniquely matched CSF transcriptome sequences showed a common distribution among NMO patients (Table 2). Surprisingly, IgG VH and VL peptides matching CSF transcriptome sequences were recovered more often in the CSF than the serum proteome. More than 50% (range 8 to 93%) of the CSF IgG transcriptome sequences were matched by peptides recovered exclusively in the CSF IgG proteome. Approximately 28% (range 7 to 46%) were found in both the CSF and serum IgG proteome, and 18% (range 0 to 69%) were found exclusively in the serum proteome. The majority of matching peptides were found exclusively in the CSF proteome in five of seven patients; in only one patient was the majority of recovered sequences found in the serum proteome (Table 2). IgG peptides matching CSF transcriptome sequences from AQP4-specific VH and VL chains showed a similar average distribution: 50% (range 0 to 100%) were recovered exclusively in the CSF IgG proteome, 25% (range 0 to 50%) in both the serum and CSF compartments, and 19% (range 0 to 50%) in the serum only. The majority of IgG peptides matching AQP4-specific VH and VL transcripts were recovered solely in the CSF in half of the patients. The low number of AQP4-specific reference sequences, however, limited this analysis (Table 2).

### Overlap between the CSF IgG proteome and blood IgG proteome

In NMO, pathogenic AQP4-IgG is presumed to passively transit into the CNS where it initiates astrocyte injury and secondary demyelination. Surprisingly, on average, only 28% of the IgG peptides matching CSF transcriptome sequences were recovered from both the CSF and serum of the same individual, which may be the result of the limited number of reference CSF transcripts. To complete a more extensive analysis of the overlap between the CSF and serum IgG proteomes, we searched the CSF and serum IgG proteomes for identical, unique VH and VL peptides independent of the transcriptome repertoires. By aligning the peptides from patients’ serum and CSF to a random human protein database (Swiss-Prot), we recovered on average 995 VH (range 613 to 1,090) and 584 VL (range 299 to 1,166) CSF and serum Ig peptides per patient. Further evaluation of these peptides for mutations from germline identified 59 unique (non-germline) IgG peptides in 5 out of 7 patients. No common unique IgG peptides were identified in different patients. In contrast to expectations, only 45% (range 0 to 77.8%) of the unique VH or VL peptides were found in both the CSF and serum compartments. Twenty-nine percent (range 11.1 to 52.9%) of the unique IgG peptides were found exclusively in the CNS compartment, 26% (range 0 to 75%) in the peripheral compartment only.

## Discussion

The detection of AQP4 autoantibodies has been shown to be a highly specific marker for NMO and AQP4-IgG plays a direct role in disease pathology. However, it is still unclear, whether CNS AQP4-IgG is primarily produced by peripheral plasma cells and passively leaks into the CNS through an open blood-brain barrier (BBB) or is generated in part by local CNS B cells. To date, serologic studies have favored a passive influx of AQP4-IgG from serum to CNS: the CSF:serum ratio of AQP4-IgG is approximately 1:500 [6] and standard metrics indicative of intrathecal AQP4-IgG synthesis are positive in only a small fraction of patients [9].

In this study, we show that the overlap between the CSF IgG transcriptome and proteome in NMO patients was found primarily, but not exclusively, within the CSF IgG fraction following acute exacerbations. These data indicate that CSF plasma cells in NMO contribute to the production of CSF AQP4-IgG and a significant fraction of the IgG idiotypes are unique to or highly enriched within the CNS compartment. The concurrent presence of many matching peptides in the CSF and serum proteomes indicates that some of the intrathecal plasma cells originate from memory B cells that had previously differentiated and populated peripheral plasma cell niches. Analyses of the overlap between the CSF and serum IgG peptides, independent of the CSF B cell transcriptomes, also reveals that a significant fraction of intrathecal IgG is unique to the CSF. Similar distributions were observed for AQP4-specific VH and VL peptide sequences. The data contrast with the expected results of a passive influx of serum AQP4-IgG, where a near complete overlap of serum and CSF IgG would be anticipated. Indeed, in our analysis, CSF metrics indicated a dysfunction of the blood-CSF barrier in only two of seven patients (Table 1).

Interestingly, approximately 20% of CSF VH transcripts matched only serum Ig peptides. The absence of overlap between these CSF B cell transcripts and the CSF Ig proteome could result from methodological limitations. While there is no data on the sensitivity and specificity of similar analyses [20,21], the high quality of the peptide sequences on mass spectroscopy and the high fidelity of the PCR transcripts lower concerns regarding low specificity. Indeed, the reproduction of functional AQP4-specific divalent IgG from the transcriptome sequences is a testament to the high specificity of the methods. In addition, it is possible that certain intrathecal B cells might not produce large amounts of IgG or their product may be sequestered in CNS tissue.

Prior studies have generated disparate results on the relationship between BBB disruption and CSF AQP4-IgG. Jarius and colleagues [10] noted an association between AQP4-IgG CSF positivity and dysfunction of the blood-CSF barrier; however, Dujmovic and colleagues [8] noted no correlation between AQP4-IgG titers and Q_Albumin_. These results suggest that the extent of BBB disruption in active NMO may not be as global as anticipated and, dependent on the lesion localization and volume, Q_Albumin_ might not be a sensitive enough marker in NMO [9]. Additionally, the entry of AQP4-IgG into the CNS may not require profound BBB permeability. While our recovery of IgG peptides matching CSF VH and VL transcripts was lower than in prior analyses in MS patients [20,21], there is no significant indication that the limited sampling skewed our results. Similar distributions were observed for both total transcripts and AQP4-specific transcripts (Table 2), and the overlap of the CSF and serum IgG proteomes demonstrated that a substantial fraction of intrathecal IgG peptides were exclusive to the CNS compartment.

**Figure 2:**
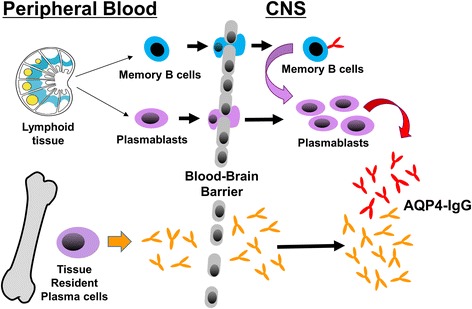
**Sources of cerebrospinal fluid (CSF) aquaporin-4 (AQP4)-IgG.** Approximately 30% of the unique intrathecal IgG peptides are specific to the CSF, while 70% are detected in serum. AQP4-IgG produced by CSF plasmablasts may contribute to the pathogenic pool of intrathecal AQP4-IgG.

The combined proteomic and transcriptome analysis of CSF IgG in NMO patients suggests that CNS AQP4-IgG is a product of both passive transit from circulating serum IgG and intrathecal plasmablasts and plasma cells (Figure 2). Approximately half of the unique germline-mutated IgG peptides in the CSF can be matched to IgG peptides in the serum, indicating that a large component of AQP4-IgG likely arises from passive transfer. Nevertheless, antibody secreting B cells in the CSF produce a measurable portion of CSF IgG and AQP4-IgG idiotypes, and the majority of those sequences cannot be matched to the serum proteome, implying an emergent population of expanded CSF B cells is present that has not populated peripheral plasma cell niches. In prior studies, recognition of the frequency of AQP4-IgG production in the CNS of active NMO patients may have been masked by the relative insensitivity of standard laboratory measures used to determine Q_IgG_ index and oligoclonal banding. These measures may also be skewed by aspects of NMO pathology (spinal cord edema and BBB dysfunction) that alter biophysical parameters such as blood-CSF diffusion and CSF flow. Furthermore, the passive transfer of serum IgG into the CSF may obscure faint bands of intrathecally produced IgG on isoelectric focusing. Finally, a significant fraction of the AQP4-IgG produced intrathecally may remain bound to glial tissue [2]. Therefore, sensitive molecular immunologic and proteomic techniques may be required to uncover the presence of intrathecal production of AQP4-IgG in most cases.

How might AQP4-IgG producing B cells and serum AQP4-IgG combine to initiate an NMO relapse? The migration of AQP4-IgG producing B cells into the CSF could be the initial step. Although AQP4 is highly expressed on perivascular astrocyte foot processes, the possibility that the limited fraction of circulating serum AQP4-IgG might initiate CNS injury is confronted by several quandaries. AQP4-specific autoantibodies are only a minor component of the total serum IgG fraction [3], CSF AQP4 titers do not always correlate with Q_Albumin_ [7], CSF AQP4-IgG is rarely detected during disease remission [9], and only 10% of NMO attacks concentrate in CNS regions with a naturally open BBB. Lastly, AQP4-IgG administered intravenously to animals fails to initiate NMO-specific pathology in the absence of autoimmune encephalomyelitis [2].

While local physiologic disturbance of the BBB due to stress or infection may precipitate an influx of a sufficient amount of circulating AQP4-IgG to induce astrocytic injury, an alternative explanation, based on our transcriptome and proteome data, is that NMO lesion formation is initiated following the transit of pathologic AQP4-IgG producing B cells to the CNS. The exclusive identification of the majority of the CSF Ig transcriptome in the CSF Ig proteome indicates that CSF plasmablasts are composed, in part, of a novel B cell population that might emerge during an acute exacerbation. In NMO CSF, plasmablasts show a remarkable degree of intraclonal diversity (reference [3] and unpublished data), which further supports recent release from germinal centers. The overlap observed between the CSF transcriptome and serum Ig proteome indicates that some intrathecal plasma cells are likely to have arisen from memory B cells that had previously established long-lived tissue-resident plasma cell clones. *In situ* production of AQP4-IgG by these transiting cells may be sufficient to produce local disruption of the BBB and facilitate passive entry of serum AQP4-IgG, lesion propagation, and clinical symptomatology.

In this study, the qualitative picture of the overlap between the CSF IgG transcriptome and the CSF and serum proteomes may have been limited by differences in the concentration and diversity of CSF and serum IgG, the small number of patients analyzed, and the timing of CSF analysis relative to relapse onset. In addition, the proportion of sequences and peptides recovered may not reflect their absolute number. Despite these caveats, the overlap of the IgG proteome and transcriptome sequences remained consistent among patients and showed evidence of both intrathecal AQP4-IgG production by CNS B cell clones and passive influx of serum IgG.

## Conclusions

In this study we show, that a fraction of intrathecal IgG including AQP4-IgG is generated by an intrathecal B cell population composed of both novel and peripherally-derived B cell clones. The production of intrathecal AQP4-IgG by CSF B cell clones indicates that future treatment in NMO may need to address both peripheral and CNS B cell populations for acute and prophylactic strategies [22-24].

## Abbreviations

ACN: acetonitrile
AQP4: aquaporin-4
AQP4-AI: AQP4 antibody index
BBB: blood-brain barrier
BCA: bicinchoninic acid
CNS: central nervous system
CSF: cerebrospinal fluid
FACS: fluorescent-activated cell sorting
IgG: immunoglobulin G
IVIG: intravenous immunoglobulin
LTQ: linear trap quadropole
NMO: neuromyelitis optica
NMOSD: neuromyelitis optica spectrum disease
PCR: polymerase chain reaction
VH: variable region heavy-chain
VL: variable region light-chain
